# Clinical Uses of Membrane Lipid Replacement Supplements in Restoring Membrane Function and Reducing Fatigue in Chronic Diseases and Cancer

**DOI:** 10.15190/d.2016.1

**Published:** 2016-02-18

**Authors:** Garth L. Nicolson, Steven Rosenblatt, Gonzalo Ferreira de Mattos, Robert Settineri, Paul C. Breeding, Rita R. Ellithorpe, Michael E. Ash

**Affiliations:** Department of Molecular Pathology, Institute for Molecular Medicine, Huntington Beach, California, 92647 USA; Saint John’s Health Center, Santa Monica, CA, USA; Laboratory of Ion Channels, School of Medicine, Universidad de la República, Montevideo, Uruguay; Sierra Productions Research, Irvine, CA, USA; Blue Hole Wellness, Wimberley, TX, USA; Tustin Longevity Center, Tustin, CA, USA; Clinical Education, Newton Abbot, Devon, UK.

**Keywords:** Membrane phospholipids, lipid oxidation, mitochondrial function, fatigue, clinical trials, chronic diseases, chronic infections, chemical contamination, neurodegenerative diseases, neurobehavioral diseases, metabolic diseases, cardiovascular diseases, metabolic syndrome, diabetes, obesity, cancer, fertility, aging

## Abstract

Membrane Lipid Replacement is the use of functional oral supplements containing cell membrane glycerolphospholipids and antioxidants to safely replace damaged membrane lipids that accumulate during aging and in various chronic and acute diseases. Most if not all clinical conditions and aging are characterized by membrane phospholipid oxidative damage, resulting in loss of membrane and cellular function. Clinical trials have shown the benefits of Membrane Lipid Replacement supplements in replenishing damaged membrane lipids and restoring mitochondrial function, resulting in reductions in fatigue in aged subjects and patients with a variety of clinical diagnoses. Recent observations have indicated that Membrane Lipid Replacement can be a useful natural supplement strategy in a variety of conditions: chronic fatigue, such as found in many diseases and disorders; fatiguing illnesses (fibromyalgia and chronic fatigue syndrome); chronic infections (Lyme disease and mycoplasmal infections); cardiovascular diseases; obesity, metabolic syndrome and diabetes; neurodegenerative diseases (Alzheimer’s disease); neurobehavioral diseases (autism spectrum disorders); fertility diseases; chemical contamination (Gulf War illnesses); and cancers (breast, colorectal and other cancers). Membrane Lipid Replacement provides general membrane nutritional support during aging and illness to improve membrane function and overall health without risk of adverse effects.

## 1. Introduction: Membrane Lipid Replacement

Membrane Lipid Replacement (MLR) is the oral supplementation of membrane glycerol-phospholipids and antioxidants to provide replacement molecules that are damaged during chronic illnesses, cancer and aging^1-4^. Replacement membrane phospholipids are important for a variety of cellular and tissue functions and for general health^2,4-6^. For example, membrane glycerol-phospholipids form the matrix for all cellular membranes and provide separation of enzymatic and chemical reactions into discrete cellular compartments and organelles, while also being essential for the function of membrane enzymes. They are also an important energy storage reservoir and provide precursors for bioactive molecules that function in signal transduction and molecular recognition pathways^7,8^. Thus MLR lipids are considered essential for general health^2,4,5^.

Orally ingested phospholipids can be degraded into their constituent parts and absorbed; they can be taken in as intact molecules without degradation, or they can be absorbed as small phospholipid droplets and micelles (reviewed in ^2^). When present in excess, most phospholipids in the gastrointestinal system are absorbed undegraded as phospholipid droplets^9^. The process is very efficient—over 90% of phospholipids are absorbed and transported into the blood within six hours^10^. In the blood circulation, they are usually found in carrier molecules, such as lipoproteins, or cell membranes of erythrocytes. Eventually they are delivered to tissues and cells where they are transferred by membrane contact and carrier or transport proteins to various cellular and organelle membranes^9^. Along this pathway, and at their ultimate destination, glycerol-phospholipids can be enzymatically modified, for example by substitution of their fatty acid side chains, to reflect the specific compositions of the membranes at their final destination. The process appears to be driven by mass action, so excess intact phospholipids have an advantage in being able to reach their final destination without degradation (reviewed in ^2^).

Patients with chronic illnesses as well as aged individuals are often deficient in MLR phospholipids, because dietary sources usually cannot provide enough MLR lipids for maintenance of undamaged cellular membranes, and thus health can suffer^2,4,5^. To provide the necessary MLR glycerolphospholipids these lipids^1-4,11^or other similar bioactive lipids^7,8^have been used as therapeutic treatments or as supporting molecules for health maintenance. Dietary lipids, such as essential glycerolphospholipids and fatty acids, are effective oral supplements, because they are successfully and efficiently absorbed in the upper small intestine within hours^2,12,13^.

In most if not all acute and chronic illnesses cellular membranes are damaged by oxidative free radicals that are produced mainly by mitochondria^2,3,14,15^. During acute and chronic illnesses the concentrations of free radical reactive oxygen species (ROS), such as superoxide anion radicals, hydroxyl radicals and hydrogen peroxide, and reactive nitrogen species (RNS), primarily peroxynitrite anion, are dramatically increased. The usual cellular antioxidants that normally neutralize these free radical oxidants are unable to neutralize all of them, and thus damage to cellular components occurs^2,3,14,15^. Membrane phospholipids and their unsaturated fatty acids are especially sensitive to oxidative damage by ROS and RNS^2,3,14^. Thus oral supplementation of membrane lipids as MLR molecules for cellular membranes and other structures has been used to restore cellular membranes^1-4,16,17^.

## 2. Compositions of MLR Lipid Supplements

The lipid compositions of most MLR supplements are not available in detail. However, the lipid composition of one MLR supplement, NTFactor^®^, was recently disclosed^4^. NTFactor Lipids^®^, a mixture of glycerolphospholipids based on mitochondrial lipid composition, contains by weight a diverse mixture of glycerolphospholipids: phosphatidylcholine (PC) 31.6%; phosphatidyl-inositol (PI) 24.9%; phosphatidylethanolamine (PE) 18.9%; phosphatidic acid 13.9%; digalactosyl-diacylglycerol 5.9%; phosphatidylglycerol (PG) 2.4%; lysophosphatidylcholine 1.0%; phosphatidyl-serine (PS) 0.5%; and monoglactosyldiacylglycerol 0.3%. It also contains by weight mainly unsaturated fatty acids: linoleic acid or 18:2D9,12 (n-6) 58.4%; palmitic acid (16:0) 19.4%; oleic acid or 18:1D9 (n-9) 9.7%; linolenic acid or 18:3D9,12,15 (n-3) 5.9%; and stearic acid (18:0) 3.9%^4^. As in all lipid extracts and from different lots, there may be some minor differences in composition from these figures.

Another MLR supplement, ‘Essential phospholipids’, has been reported to contain 76% PC, 7% PE, 0.5% PI and other undisclosed lipids^14,15^. Approximately 70% of its fatty acids are linolenic acid^17^.**

## 3. MLR Lipid Supplements are Safe

Extremely high doses of MLR phospholipids have been given to animals and humans with no apparent acute or chronic toxicities (reviewed in ^2,17^). For example, in a long-term phase I/II clinical trial using patients with cardiovascular disease, PI was given at doses over 5 g per day with no adverse effects^18^. The PI was shown to increase plasma high-density lipoprotein-cholesterol and apolipoprotein A1 levels and reduce triglyceride levels without any evidence of toxicity^18^.

The use of relatively high oral doses of replacement membrane lipids has actually improved cardiovascular blood markers. In one study, older subjects (60.7 average age) received over 2 g per day of oral glycerolphospholipids (NTFactor^®^) for over 6 months and showed no adverse effects of the MLR phospholipids. In fact, cardiovascular blood marker levels, such as homocysteine, improved significantly during this period (p<0.001) without any evidence of any adverse effects from the MLR phospholipids^19^. In fact, there has been no evidence of any toxicity or adverse events from the long-term use of MLR glycerolphospholipids^2,4,11,16,17,19,20^. Therefore, the U.S. Federal Drug Administration (FDA) has classified MLR phospholipids as ‘Generally Recognized as Safe’ or GRAS^21^.

## 4. Development of Various MLR Lipid Supplements

Various oral dietary phospholipid supplements have been developed over the years that contain mixtures of the common membrane glycerolphospholipids and other lipid components^1,2,4,11,16,17^. Of the commercially available oral phospholipid supplements, most are lecithin preparations that lack protection from oxidation and enzymatic destruction^2,17^. However, some oral MLR supplements, such as NTFactor^®^ and NTFactor Lipids^®^, are protected against oxidation and degradation and fulfill the requirements for convenience, efficacy and safety^1,4^. The MLR lipids in NTFactor^®^ and NTFactor Lipids^®^ contain fructooligosaccharides that protect the glycerolphospholipids from oxidation, bile and enzymatic destruction and temperature extremes^22^. Daily use of MLR supplements have been found to be safe in amounts of several grams of glycerolphospholipids per day^2,4^.

Recently supplements have been developed that contain MLR phospholipids along with additional non-MLR lipid ingredients. For example, some NTFactor lipid-containing supplements also contain probiotic bacteria, various vitamins and minerals and other components (Propax^TM^ with NTFactor^®^, Advanced Physician’s Formula^TM ^, Healthy Aging^TM^, Pro-Green Energy with NTFactor^®^, among others). A specific supplement for mitochondrial support, ATP Fuel^®^^23^, contains NTFactor^®^ and also NADH, Coenzyme-Q10, vitamin E, a-ketoglutaric acid, L-carnitine, and other ingredients in order to overcome mitochondrial deficiencies other than damaged membrane phospholipids^24^. Oral MLR products, such as those containing purified phosphatidyl-serine (PS), are also commercially available^25^.

An intravenous-delivered MLR mixture of glycerolphospholipids (‘Essential’ Phospholipids) has been developed that can deliver high concentrations of MLR phospholipids without the need for protective fructooligosaccharides^17^. However, this product is still susceptible to enzymatic and oxidative damage during storage, and daily intravenous delivery comes with some risk for adverse events, such as infection, blood vessel damage, and other potential problems^4^.

## 5. Mitochondrial Function and Fatigue

The most common clinical use of MLR supplements is to treat fatigue^1-4^. Fatigue is the most common complaint of patients seeking general medical care, and it is associated with aging and most if not all chronic medical conditions^26,27^. Fatigue is considered a complex, multi-dimensional sensation that is not well understood but is perceived to be a loss of overall energy, mental or physical tiredness, a feeling of exhaustion or diminished endurance, and an inability to perform even simple tasks without exertion^27,28^. Fatigue develops during aging and chronic diseases due to a variety of causes, including loss of mitochondrial function^24,28,29^. Indeed, moderate to severe fatigue has been directly related to loss of mitochondrial function and diminished production or leakage of ATP^24,28,30^.

Some clinical conditions associated with fatigue are closely related to loss of mitochondrial function. Fatigue in chronic fatigue syndrome (CFS/ME), fibromyalgia, cancer, among other conditions, is clearly related to mitochondrial dysfunction, whereas other disorders are less clearly associated with loss of mitochondrial function. For example, certain mental conditions, such as schizophrenia, bipolar disease, among others, some cancers and other conditions may or may not be associated with mitochondrial dysfunction^28^. Mental and cancer patients are often characterized with moderate to severe depression, and depression is frequently related to mild fatigue states where it shows some physical, cognitive and emotional overlap with fatigue^31^.

Loss of mitochondrial function has been related to oxidative damage to mitochondrial membrane lipids^22,24,28,32^. Inner mitochondrial membrane (MIM) lipid damage increases proton and ion leakiness and lowers trans-membrane potential of the MIM, reducing the production of ATP. The consequence of a loss in ATP production is reduced physical and mental performance, as seen with aging and disease^24,29,30,32^. This is obviously seen in chronic fatigue syndrome patients, as these patients present with evidence of oxidative damage to their DNA and lipids, such as oxidized blood markers and oxidized membrane lipids^32-35^. These are indicators of excess oxidative stress^33-35^. Thus patients have sustained, elevated levels of free radical oxidants, such as ROS and RNS, which can result in loss of mitochondrial function as well as changes in cytokine levels that can exert a positive feedback on free radical production^34^.

## 6. Use of Oral MLR Lipid Supplements to Treat Fatigue

MLR supplements have been used in several clinical studies designed to treat moderate to severe chronic fatigue in patients in order to reduce their fatigue scores^4,36-42^ (**Table 1**). Although most of the clinical studies in**Table 1** were relatively small (less than 50 participants) and open-label, there were significant outcomes in terms of fatigue reductions. One non-open label exception was a cross-over trial studying the effects of oral NTFactor^®^ on fatigue in moderately to severely fatigued subjects^37^. In this cross-over trial there was good correspondence between reductions in fatigue and gains in mitochondrial function, as assessed by MIM redox potential. After 8 weeks of MLR with 4 g per day of NTFactor^®^, mitochondrial function improved significantly, and after 12 weeks of NTFactor^® ^supplementation, mitochondrial function was found to be similar to that found in young healthy adults (26.8% increase, p<0.0001). In this study fatigue was reduced 35.5% (p<0.001) during this period^37^. After 12 weeks of supplement use, subjects were placed on placebo without their knowledge for an additional 12 weeks, and their fatigue and mitochondrial function were measured. After the 12-week placebo period, fatigue and mitochondrial function were intermediate between the initial starting values and those found after eight or 12 weeks on the supplement^37^. Similar results on the effects of NTFactor on fatigue were found in patients with chronic fatigue syndrome (CFS/ME) and/or fibromyalgia, Gulf War illness and chronic Lyme disease (reductions from 26-43%,**Table 1**). Analyzed together, these clinical data indicate that MLR can be successfully used to reduce fatigue in patients with different diagnoses and moderate to severe fatigue.

Loss of mitochondrial function is not always exclusively linked to membrane lipid damage^24^. To overcome this problem supplements containing coenzyme Q10, L-carnitine, alpha-lipoic acid, NADH, along with MLR glycerol-phospholipids, have been used as combination mitochondrial supplements. As an example of a combination MLR supplement, ATP Fuel^®^ has been used to treat long-term chronic illness patients with intractable fatigue^23,41,42^. The patients in the ATP Fuel^®^ clinical studies had been ill with moderate to severe intractable fatigue for an average of over 17 years, and they had been seen by multiple physicians (>15). In addition, they had taken an average of over 35 supplements or drugs with no effect on their fatigue^23,42^. These patients responded to the MLR combination supplement, showing significant reductions in fatigue within 6 weeks (p<0.0001). Regression analysis of the fatigue data indicated that the reductions were gradual, consistent, and occurred with a high degree of confidence (R^2^ = 0.960-0.998). Thus, the MLR combination supplement proved to be a safe and effective method to significantly reduce fatigue in patients, even after years of intractable fatigue^23-41^.

**Table 1:** Clinical trials and effects of NTFactor® MLR supplements on fatigue scores. ^a^Modified from Nicolson^3^; ^b^Piper Fatigue Scale^43^; ^c^Advanced Physician’s Formula with NTFactor^®^; ^§^p<0.0001, *p<0.001 t-test with/without NTFactor^®^; Av. - Average; CFS - chronic fatigue syndrome; ME - myalgic encephalomyelitis; GW – Gulf War;

MLR Supplment	Subjects/patients	No. of patients	Av. age Time (weeks)	Reduction (%)b	Reference
Propax/ NTFactor^®^	Chronic fatigue	34	50.3	40.5 ^§^	Ellithorpe et al.^36^
NTFactor^®^	Aging, chronic fatigue	22	68.9	35.5 *	Agadjanyan et al.^37^
NTFactor^®^	CFS/fibromyalgia	15	44.8	43.1 *	Nicolson & Ellithorpe^38^
Healthy Curb^®^	Obesity, fatigue	35	42	23.9 *	Ellithrope et al.^39^
APF/ NTFactor^®c^	Aging, chronic fatigue	67	57.3	36.8 ^§^	Nicolson et al.^40^
Propax/ NTFactor^®^	Cancer, fatigue	35	50.7	30.1 *	Nicolson^3^
ATP Fuel^®^/ NTFactor^®^	CFS/ME	30	55	30.7 ^§^	Nicolson et al.^23,41^
ATP Fuel^®^/ NTFactor^®^ GW	GW Illness, fatigue	16	42.2	34.6 *	Nicolson et al.^23^
ATP Fuel^®^/ NTFactor^®^	Lyme disease, fatigue	17	52.4	26.0 *	Nicolson et al.^42^

## 7. MLR Oral Lipid Supplements and Cancer

In addition to fatigue in chronic illnesses, fatigue is one of the most common symptoms found in cancer patients. It occurs in the earliest forms of cancer to the most progressed metastatic states^3^. Cancer-associated fatigue is not well understood, but it is thought to be due to a combination of the effects of cancer itself plus the effects of cancer treatments^3,44^. MLR supplements have been used to treat cancer-associated fatigue and the fatigue and other side effects of cancer therapy^3,44,45^. For example, Propax^TM^ with NTFactor^® ^has been used to reduce some of the adverse effects of cancer therapy, such as chemotherapy-induced fatigue, nausea, vomiting, malaise, diarrhea, headaches and other adverse effects of therapy^45^. In advanced metastatic colon, pancreatic and rectal cancer patients Propax^TM^ with NTFactor^® ^reduced the adverse effects of chemotherapy, decreasing significantly episodes of severe fatigue, nausea, diarrhea, constipation, skin changes, insomnia and other effects, as assessed by nurses and by the patients themselves. For example, 81% of the patients on chemotherapy that used Propax^TM^ with NTFactor^®^ experienced overall improvements in quality of life parameters. In a subsequent double-blind, placebo-controlled cross-over trial containing 36 patients on chemotherapy plus Propax^TM^ there were fewer adverse effects of therapy, resulting in improvements in fatigue, nausea, diarrhea, impaired taste, constipation, insomnia and other quality of life indicators^45^. This indicates that MLR supplements can improve cancer care by reducing adverse complications of treating cancers as well as reducing cancer-associated fatigue^3^.

**Table 2:** Suggested Doses, Current and Potential Uses of Oral MLR Supplements ^a^Dose range in grams per day based on weight of NTFactor Lipids^®^^b^NTFactor^®^ or NTFactor Lipids^®^; CD – cardiovascular disease; CFS/ME – chronic fatigue syndrome/myalgic encephalomyelitis; GW – Gulf War; MetSyn – metabolic syndrome;

Use	Subjects/patients	Age group	MLR Lipid Supplement	NTFL Dose^a^ range (g/day)	Reference
General health	Aged	senior	NTFactor/L^b^	2-3	Ellithorpe et al.^36^
Fatigue	Aged	senior	NTFactor/L	2-3	Agadjanyan et al.^37^
Fatigue	CFS/ME	adult/teen	NTFactor/L	2-4	Nicolson & Ellithorpe^38^
Fatigue	CFS/ME	adult	ATP Fuel	4	Nicolson et al.^23,41^
Fatigue	Fibromyalgia	adult	NTFactor/L	3-4	Nicolson & Ellithorpe^38^
Weight loss	Obesity, fatigue	adult	NTFactor	1-3	Ellithrope et al.^39^
General health	Neurodegenerative Dis.	adult	NTFactor/L	3-4	Nicolson et al.^46^
CD health	CD risk/CD disease	adult	NTFactor/L	2-4	Ellithorpe et al.^36^
Metabolic health	MetSyn/diabetes	adult	NTFactor/L	2-4	Nicolson^47^
Metabolic health	Diabetes	adult	ATP Fuel	4	Nicolson et al.^23^
Neurobehavior	Autism Spectrum Dis.	child	NTFactor/L	1-2	Nicolson et al.^46^
Infections	Lyme/mycoplasma	adult	ATP Fuel	4	Nicolson et al.^42^
Fertility	Fertility Diseases	adult	NTFactor/L	2-3	-
Fatigue	Cancer	adult	NTFactor/L	2-3	Nicolson and Conklin^44^
Anemia	Anemia	adult	NTFactor/L	1-2	Ellithorpe et al.^36^
Injury	Spinal injury	adult	NTFactor/L	1-2	Ellithorpe et al.^36^
Autoimmune	Rheumatoid arthritis	adult	ATP Fuel	4	Nicolson et al.^23^
General health	Pregnancy	adult	NTFactor/L	1-2	Ellithorpe et al.^36^
Chemical detox	GW Illnesses	adult	NTFactor/L	>4	Nicolson et al.^48^
Mental clarity	Aging	adult	NTFactor L	1-2	Ellithorpe et al.^49^

## 8. Other Clinical Uses of Oral MLR Supplements

MLR lipid supplements have been used in a variety of clinical disorders (**Table 2**). For example, MLR may modify the metabolism through body mass reduction and appetite restraint^39^. In this study 30 obese but otherwise normal subjects took oral HealthyCurb^®^, aNTFactor^®^ and alpha-amylase inhibitor formulation, 30 min before each meal. Over time subjects gradually lost weight, as shown by significant reductions in weight and waist and hip circumferences. They also showed reductions in body mass index (BMI) and basal metabolic rate (BMR) (p<0.001). Overall hunger was reduced during the trial by 44.5% (p<0.001), with decreased cravings for sweets and fats. There was also a 23.9% reduction in fatigue (p<0.009). Along with fatigue reduction, subjects had a 26.8% enhancement in perceived improvements in cognition and increased abilities to concentrate, remember and think clearly (p<0.004)^39^.

Improvements in mental clarity while on MLR lipid supplements have been examined in a preliminary study using NTFactor Lipids^®^^49^. A group of 29 subjects were given 0.6 g of NTFactor Lipids^®^ in a drink, and fatigue and mental focus were surveyed after three hours. At this time a self-reported survey instrument was given to the participants. A majority of the participants responded within one hour, and by three hours they reported improvements in cognition, mental clarity and focus along with reductions in perceived fatigue^49^.

Instead of combinations of glycerol-phospholipids, specific single glycerol-phospholipids, such as oral PS, have been used in clinical studies to assess improvements in memory and cognition. In such a study 30 male and female subjects aged 50-90 years were enrolled with memory impairments unrelated to neurological disease, stroke, diabetes, infections or inflammatory processes. After 12-weeks on 300 mg oral PS per day the outcomes in six different tests of memory and cognition were determined^50^. At the end of the trial participants showed significant improvements in memory recognition (p=0.004) and recall (p=0.006), total learning (p=0.013), executive functions (p=0.004), metal flexibility, and visual spatial learning. There were no adverse events during the trial, and interestingly both mean systolic and diastolic blood pressure values were reduced at 12 weeks in comparison with baseline values^50^. A similar double-blind, randomized clinical trial on 78 subjects (50-69 years old) was conducted to determine if 100-300 mg oral PS per day versus placebo improved memory scores. In this study, PS significantly improved behavioral memory functions, especially short-term memory and cognitive function in low-scoring (delayed word recall) participants (p<0.01)^51^.

MLR lipid preparations have been used to treat memory loss in aged subjects and in Alzheimer’s disease (AD)^50-52^. AD patients were supplemented with 300 mg per day PS for 6 months. At the end of this period participants showed cognitive improvements relative to placebo controls^52^. This result, however, was not found in another study on elderly subjects with age-associated memory impairment that received 300-600 mg soy PS daily for 12 weeks^25^.

## 9. Final Comments

Oral MLR lipid formulations have proven to be safe and effective as daily supplements for replacing damaged membrane glycerolphospholipids and restoring membrane function. That replacing damaged membrane phospholipids has a positive effect on cellular function and health, such as mitochondrial function, has been demonstrated in clinical trials. The positive effects of MLR lipids are also apparent in other membrane compartments, such as the plasma membrane, and additional studies will undoubtedly concentrate on the functional effects of MLR lipids on the plasma membranes of various cell types. Since MLR natural lipid supplements are not drugs, the usual drug safety issues do not apply, as extremely high doses of MLR glycerolphospholipids have been given to animals and humans over long periods of time without any evidence of toxic or adverse effects^2^.

It should be clear that additional clinical studies are needed, especially controlled clinical trials, to further examine the role of MLR lipid supplements in improving health and reducing symptoms like fatigue. Important future development will likely center on the neurological aspects of aging and illnesses, such as mood, cognition, executive function, mental flexibility and learning. Also, the long-term use of MLR lipid supplements to enhance cardiovascular health will be another important area of research.

The daily use of MLR lipid supplements to improve health during aging and support the treatment regimens for various clinical conditions is completely justified because of their efficacy and safety. New formulations of MLR lipids and combination MLR supplements are being developed, and these will eventually allow the lipid replacement and repair of vital cellular membranes and other cellular structures that are damaged during aging and illness.
